# An Evaluation of Microbial and Chemical Contamination Sources Related to the Deterioration of Tap Water Quality in the Household Water Supply System

**DOI:** 10.3390/ijerph10094143

**Published:** 2013-09-06

**Authors:** Yoonjin Lee

**Affiliations:** Department of General Education, Konyang University, 121 Daehakro, Nonsan, Chungnam 320-711, Korea; E-Mail: leeyj@konyang.ac.kr; Tel.: +82-41-730-5747; Fax: +82-41-730-5526

**Keywords:** drinking water, biofilm, volatile organic compounds, water supply systems, household plumbing

## Abstract

The predominant microorganisms in samples taken from shower heads in residences in the Korean city “*N*” were *Stenotrophomonas maltophilia*, *Sphingomonas paucimobilis*, *Acidovorax temperans*, and *Microbacterium lacticum*. *Legionella* was not detected in this case. The volatile organic compounds (VOCs) vinylacetate, NN-DMA, *cis*-1,2-dichloroethylene, epichlorohydrin, and styrene were measured in five types of plastic pipes: PVC, PB, PP, PE, and cPVC. The rate of multiplication of the heterotrophic plate count (HPC) attached on the copper pipe in contact with hot tap water was higher than the rate for the copper pipe in contact with cold tap water. Biofilm accumulation on stainless steel pipes with added acetate (3 mg/L) was 2.56 times higher than the non-supplemented condition. Therefore, the growth of HPC in the pipe system was affected by the type and availability of nutrients and depended on variables such as heating during the hot water supply.

## 1. Introduction

Tap water treated in water plants becomes exposed to various pollutant sources before it reaches the consumer’s faucet. The management of a water distribution system and a water treatment process is regarded as important to the supply of quality tap water. Even when disinfection occurs during water treatment processes, bacterial regrowth may occur in the long journey through water distribution systems. Yoon and Lee reported that the level of residual chlorine decreased at the endpoints of Korean water treatment systems [1]. Bacterial regrowth in water distribution systems can occur because of the accidental entry of microorganisms at cross connections and broken pipes and the recovery of microorganism populations affected by disinfectants in water treatment plants.

The formation of a biofilm on the surface of pipes is generally believed to be responsible for the deterioration of microbial water quality and the onset of disease via pathogen release [2]. Opportunistic pathogens and pathogens that grow in biofilms include *Escherichia coli*, *Pseudomonas*, *Mycobacteria*, *Campylobacter*, *Klebesiella*, *Aeromonas*, *Legionella* spp., *Helicobacter pylori*, and *Salmonella typhimurium* [3,4,5,6]*.* A biofilm is formed by the following multi-step process: initiation, maturation, maintenance, and dissolution. Siller *et al.* defined a biofilm as a dense aggregate of surface-adherent microorganisms embedded in a polysaccharide matrix [7]. The important factors influencing biofilm formation are temperature, pipe materials, residual disinfectants, nutrients in water, and hydraulic conditions in water distribution systems [8].

Because the safety of drinking tap water is still a concern, many domestic consumers purchase bottled water or a private filtration system [9]. In Korea, several provincial governments have acknowledged that consumers have little confidence in the safety of tap water and have sometimes even supplied free bottled tap water to the public at official government events. Various factors are involved in obtaining a high quality of tap water for the consumer, including aesthetics, such as taste and odor, in addition to the prevention of waterborne diseases and the removal of harmful chemicals. Water supply systems consists of various components, including pipes, valves, and water reserve tanks. Park *et al.* reported that the inspection of water quality in water tanks was not regularly performed for 175 out of 515 locations in Kyungnam, Korea, and the heterotrophic plate counts (HPCs) were over 100 colony-forming units (CFU) at 27 locations [10]. Lee *et al.* concluded that water quality deterioration, lower water pressure, and leaks were observed among service pipes over the 10 years of their water supply period [11].

Metal piping, such as copper, is conventionally used in home plumbing systems. However, polymer pipes, such as cross-linked polyethylene (PEX), polyethylene (PE), and polyvinyl chloride (PVC), are increasingly used in water supply systems. Polymer pipes are cost effective and easy to install. However, the substances leached from plastic pipes could cause health-related and aesthetic problems, including undesirable taste and odor. The amount of PE and PVC pipes used in the water supply system in South Korea is reported at 48%. Various plastic components, such as pipe valves, reserve tanks, and joints, are being utilized in water supply systems. Nam and Lee reported that compared to copper piping, plastic pipes cause a greater multiplication of microbial organisms [12]. Coliform bacteria contaminated drinking water in a system that used rubber-coated valves in a German water supply system [13]. The migration of organotin and chlorinated compounds was reported in PVC piping [14].

This study evaluated the micro-chemical contamination of water facilities in water supply systems. Samples of the predominant microorganisms were taken from residential shower heads, and the growth characteristics of biofilms in the water supply system were analyzed. The multiplication of microorganisms and their nutrient sources were studied, and methods for controlling microbial regrowth in different water system materials were compared. In addition, volatile organic compounds (VOCs) and heavy metals leached from plastic pipes were evaluated in this study.

## 2. Materials and Methods

### 2.1. Feed Water

Samples of tap water were taken from the Engineering Department of Konkuk University, Seoul, Korea. These samples were used as the source material of water that was fed into the plumbing system. The tap water had been conventionally treated by chemical coagulation with polyaluminum chloride, sedimentation, filtration, and disinfection with free chlorine at the Guui water treatment plant. The tap water quality data is presented in Table 1. Residual chlorine was almost depleted in hot water, and dissolved organic carbon (DOC) and UV_254_ were similar in both conditions. The source of assimilable organic matter (AOC) was sodium acetate. The sources of inorganic matter were KNO_3_, KH_2_PO_4_, Na_2_SO_4_, Na_2_SO_4_, CaCl_2_·2H_2_O, MgCl_2_·2H_2_O, FeCl_3_·6H_2_O, CoCl_2_·6H_2_O, CuCl_2_·2H_2_O, MnSO_4_·5H_2_O, FeCl_3_·6H_2_O, CoCl_2_·6H_2_O, MnSO_4_·5H_2_O, ZnCl_2_, and (NH_4_)_6_Mo_7_O_24_·H_2_O. A stock solution (500 mg/L) of sodium acetate and an inorganic nutrient cocktail in sterile deionized water were initially prepared and diluted with sterile deionized water to the concentrations of acetate and inorganic nutrients needed in each experiment.

**Table 1:** Tap water quality used in this study.

Items	Cold water	Hot water
DOC (mg/L)	1.5 ± 0.1	1.4 ± 0.1
Turbidity (NTU)	<0.4	<0.4
pH	7.1 ± 0.05	7.4 ± 0.05
Conductivity (µS/cm)	180 ± 5	180 ± 5
Residual chlorine (mg/L)	<0.3	<0.1
UV-254	0.009 ± 0.001	0.010 ± 0.001
DO (mg/L)	10 ± 2.5	10 ± 2.5

### 2.2. Pipe System

**Figure 1:**
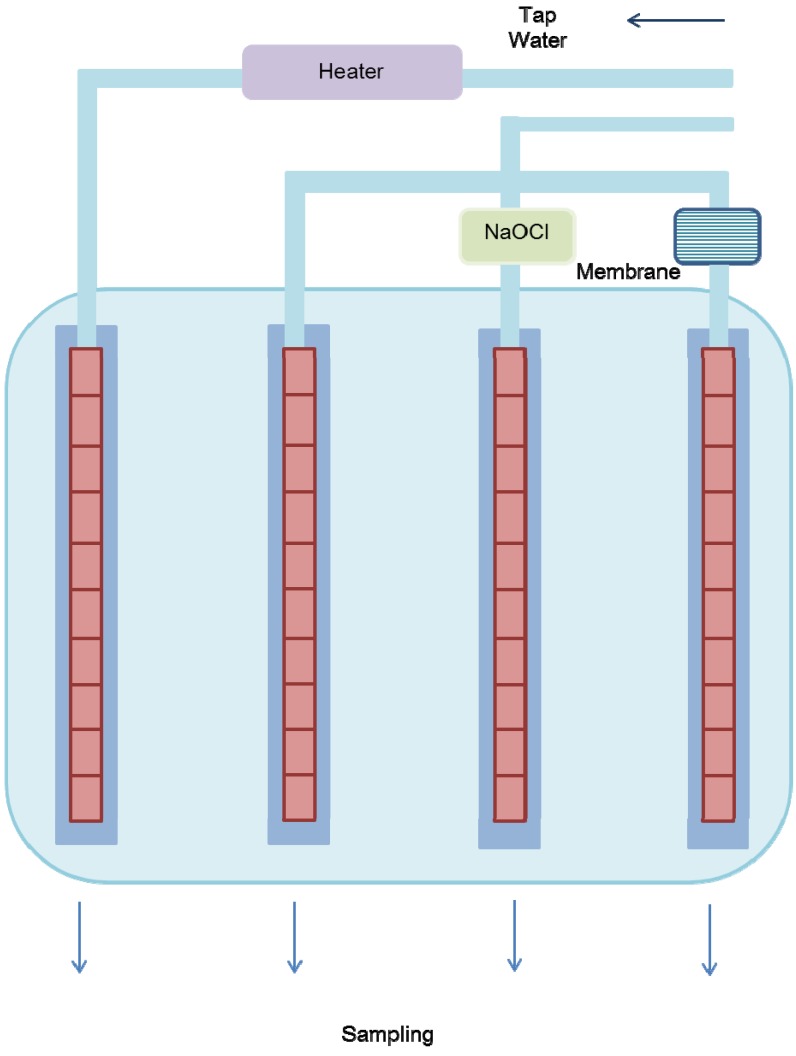
Schematic diagram of the experimental system.

PEX, polybutylene (PB), polypropylene pipes (PP), PE, PVC, and chlorinated polyvinyl chloride (CPVC) were selected in this experiment to determine VOC leaching. The PVC, PEX, copper, and stainless steel pipes used for the analysis of biofilm formation were cut into 71-mm sections with a stainless steel cutter and were washed with a sonicator before setting. The pipe units were composed of 10 pipe sections. Water temperatures in the system were maintained at 20 °C and 55 °C (Figure 1). The feed water was changed every 7 days. After the desired time was reached, the pipe sections were withdrawn for analysis. The first biofilm sampling took place at 120 days from the start of the systems operations, and sampling was repeated every 60 days until the end of the test period. In the second experiment, bacterial multiplication in the pipes was observed with chlorination and membrane filtration. Chlorinated water was produced with sodium hypochlorite (5%, Wako, Tokyo, Japan), and water was filtered through a 0.45-μm membrane (Millipore, Bilerica, MA, USA). Bacterial multiplication and adenosine triphosphate (ATP) were monitored in the presence of chlorination and membrane filtration at 12, 30, 60, and 90 days.

### 2.3. Chemical Analysis

All analyses were based on standard methods for the examination of wastewater and drinking water in Korea [15,16]. DOC was analyzed using a total organic carbon (TOC) analyzer (TOC 5000, Shimadzu, Kyoto, Japan) after filtering with a 0.45-µm pore size membrane filter. Turbidity was analyzed using a turbidity meter (Hach 2100, Loveland, CO, USA) and is presented in NTU units. Metal substances were measured by ICP (Inductively Coupled Plasma Spectroscopy, Labtam 8440, Braeside, Austrailia).

Residual chlorine levels were determined at the time of collection using the N,N-diethyl-*p*-phenylenediamine (DPD) procedure. The VOC determination process followed. Domestic brands of PB, PE, PVC, and PP pipes were selected for the test. The test procedure was based on the Korean standard methods for drinking water. The inner diameters of PB, PE, PVC PP, CPVC pipes were 20, 21, 28, 25, and 26 mm, respectively. The length of each test pipe was 93 mm. The test pipes were in contact with 500 mL of Milli-Q water for 24 h. The migration tests for plastic pipes were performed in triplicate. The analysis was conducted according to standard methods used for drinking water in Korea. Water samples and reference samples were concentrated using a purge and trap method. VOCs were determined with a Hewlett Packard 6890 gas chromatograph (Avondale, PA, USA).

### 2.4. Bacterial Analysis

Each plate was inoculated with 0.1 mL of a sample, and the plates were then incubated at 20 °C for five days. All bacterial counts were performed in triplicate. Deionized water was sterilized in an autoclave, and 5 mL was aliquoted in tubes. Biomasses on pipes were taken from the surfaces with sterilized swabs, which were placed into the tubes and mixed in a vortex mixer for 2 min (KMC-1300, Vision Scientific, Bucheon, Korea). The prepared samples were inoculated using the spread-plate method on R2A plates. The colonies were counted and presented as CFU per cm^2^. ATP levels were determined using an ATP analyzer (AMSA lite ΙΙ, Auburn, MI, USA).

Bacterial analysis was performed for shower samples taken from a residential area in the city of “*N*”. The samples were preserved in sterilized plastic bags until they reached the laboratory, where the samples were then stored in a refrigerator at 4 °C. The shower head was disassembled from the mains, and samples of the biomass were taken from inside the shower head with sterilized swabs soaked with distilled water. The biomass samples were spread on buffered charcoal yeast extract agar (Sigma D3560, St. Louis, MO, USA). Inoculated plates were incubated in a CO_2_ incubator at 35 °C for 7 days. The colonies formed on these plates were cultivated for pure culture isolation. Bacteria selected by observation with the naked eye were identified by 16S ribosomal DNA analysis. Chromosomal DNA was extracted and purified according to Sambrook *et al.* [17]. The 16S rRNA genes were amplified by PCR with the universal primers 27F and 1492R and were the sequenced using an ABI prism BigDye terminator cycle sequencing ready-reaction kit and an ABI 3730xl DNA Analyzer. The amplified 16S rRNA gene sequences of the isolates were collected using the SeqMan software (DNASTAR, Madison, WI, USA). The 16S rRNA gene sequences of the isolates were analyzed with available 16S rRNA gene sequences from GenBank using the BLAST program and the EzTaxon server to determine the phylogenetic affiliations [18].

## 3. Results and Discussion

### 3.1. Biofilm Formation in Hot Water Plumbing

**Figure 2:**
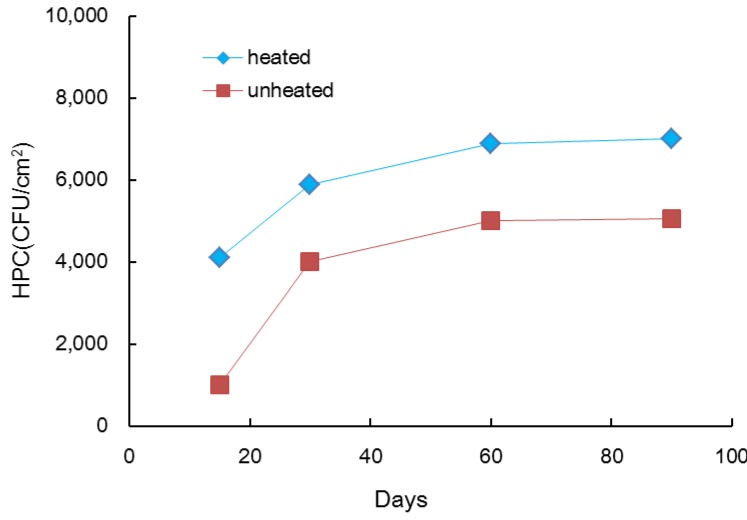
Biofilm accumulation, as assessed with HPC on PEX pipes in contact with heated and non-heated tap water.

In this experiment, the effect of heated tap water in contact with PEX pipes was observed (Figure 2). The level of microorganisms that grew on PEX pipes with heated water was 1.4 times higher at 90 days than the level in unheated conditions. This observation could be explained by the change in nutrient availability after the tap water was heated. The biofilm HPC increased over time; and the HPC had nearly reached its maximum level by 30 days. However, the extent of the increase in the HPC in heated conditions was greater in the PEX pipe than in unheated conditions. PEX pipe is often used in Korean hot water plumbing. Skjevrak *et al.* concluded that biofilm in pipes may function as a reservoir for potent VOC odors associated with the algae and cyanobacteria that exist in the raw water source. The VOC amounts were slightly higher in new HDPE pipes compared to HDPE pipes used at the beginning of the test [19].

**Figure 3:**
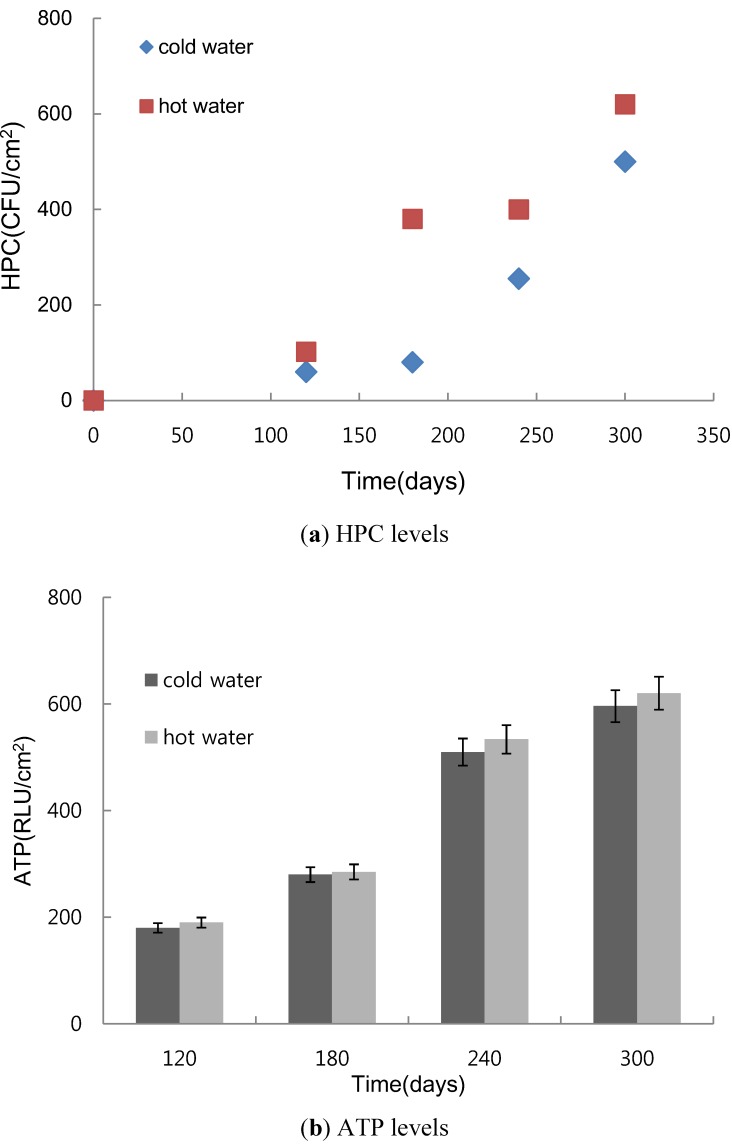
Variations in attached HPC and ATP levels on copper pipes per unit surface area in contact with both cold and hot water from a building-hold faucet.

Higher HPCs were detected on copper pipes with hot tap water compared to the faucet that provides cold water (Figure 3(a)). Temperatures were kept constant at 20 °C and 45 °C for the respective cold water and hot water copper pipes during this experiment. It is generally known that the growth rate of microbes increases two- to three-fold over an increase of 10 °C between the minimum and optimum growth temperatures. The biofilm HPCs on copper pipe increased over time and was 1.2 times higher for hot water than for cold water at 300 days. Lee reported that pipe material and temperature affect bacterial growth in water distribution systems [20].

The ATP levels increased over time in copper pipe, as shown in Figure 3(b). The ATP levels at 45 °C were higher than the levels at 20 °C, though the difference was not significant. The ATP levels varied with pipe material and were higher in stainless steel pipes than in copper, carbon steel, and galvanized pipes [21]. The quality of tap water already properly treated in water treatment plants could be polluted within a hot water supply system for various reasons. Nam and Lee reported higher microorganism levels at 55 °C compared to 20 °C in effluents from PEX pipe [12]. Borella *et al.* reported that the distribution of *Legionella* differed according to heater type, water temperature, and free chlorine because of different sensitivities and resistance levels to environmental factors [22]. In this study, the biofilm HPCs on copper pipe consistently increased until 300 days. ATP levels at 300 days were 3.2 and 3.3 times higher than the respective levels at 180 days for cold and hot water. Another study concluded that major risk sources for Legionnaire’s disease were located in old buildings [23].

**Figure 4:**
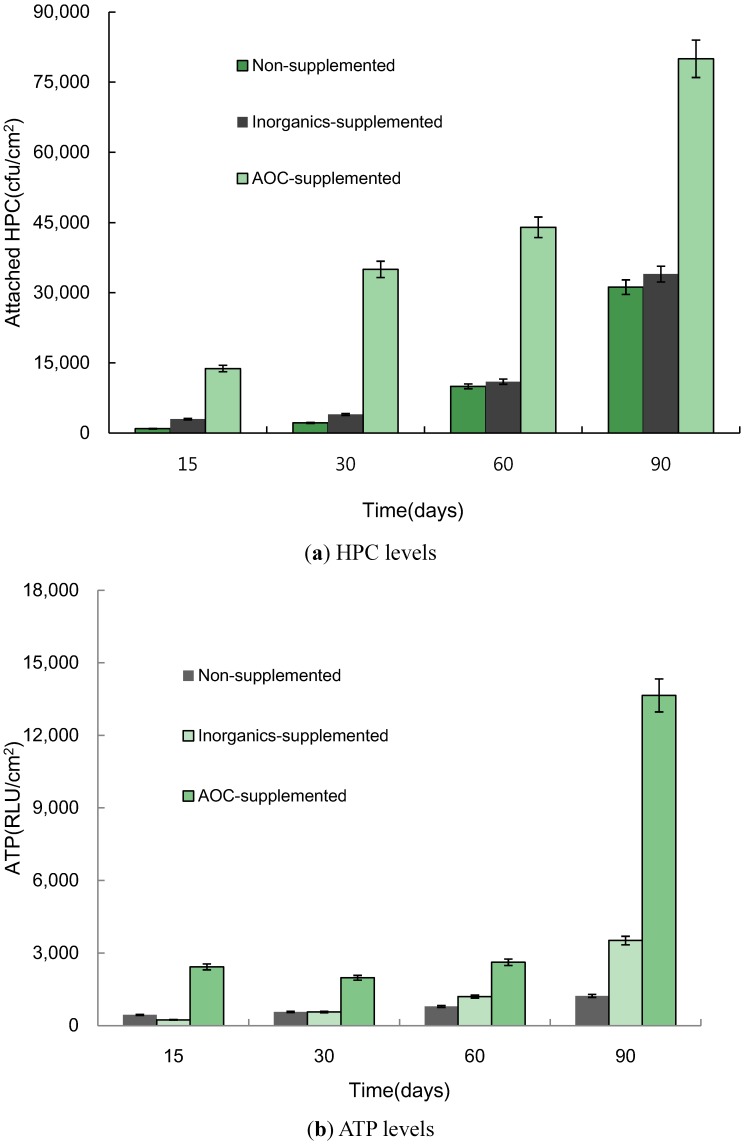
Biofilm accumulation on stainless steel pipe, as assessed with HPC and ATP level, on PEX pipes in the control experimental condition and with the addition of 3 mg/L acetate and an inorganic cocktail in tap water.

Microbial growth on stainless steel pipes increased enormously with the supply of 3 mg/L sodium acetate (Figure 4). The HPC for 3 mg/L sodium acetate at 90 days was 2.6 times higher compared to the control. Lee reported that total coliform bacteria counts in samples correlated with DOC levels in 80 water samples taken from Korean spas [24].

### 3.2. Effect of the Addition of Nutrient Sources on Biofilm Formation

Attached HPCs at 90 days were 32.8, 11.3, and 5.8 times higher than HPCs at 15 days in controlled, inorganic, and AOC-supplemented conditions, respectively. In this experiment, the HPC on stainless steel with the addition of an inorganic cocktail was higher compared to the control. Previously, we observed that the contribution of inorganic nutrients was significant in the Korean water distribution system and that phosphorous in particular was a limiting substance in PVC pipes [12]. Biofilm accumulation, as assessed by HPCs on stainless steel pipe, increased with the addition of inorganic substances in this study and was observed to increase over time. The ATP levels showed the same tendency as displayed by HPCs. The ATP level in the inorganic supplement was 2.9 times higher compared to the control at 90 days. The ATP levels in the biofilms at 90 days were 2.8, 15, and 5.6 times higher than the levels at 15 days for the non-supplemented, inorganic, and AOC-supplemented conditions. In this experiment, carbon was regarded as the main factor affecting microbial regrowth in stainless steel piping.

The results indicated that the accidental inlet of nutrient sources during the transportation of tap water to the consumer, such as cross-connections with sewer lines, could cause microbial regrowth as well as biofilm growth in the water pipe systems. Appenzeller *et al.* reported a clear relationship between bacterial growth and phosphate levels, using 0.1 to 2 mg P-PO_4_/L [25]. However, Nam and Lee reported that 0.5 mg/L of P in PEX pipe crucially affected the multiplication of HPC [12]. The results indicated that the efficiency of nutrient removal, including carbon in water treatment plants, and the level of change in nutrient availability throughout the water distribution were related to biofilm growth on pipes in the water supply system. Lee reported that the addition of a phosphate inhibitor in tap water increased the number of microorganism on the surfaces of PEX pipes [20]. The HPC for PVC pipe with phosphate inhibitor was 2.5 times higher compared to the untreated condition [20]. Hence, the nutrient source in tap water was shown to be an important factor in the accumulation of microbes attached to water pipe systems. ATP values exhibit the same trend as HPCs.

### 3.3. Multiplication of Microorganisms on Shower Heads

Microbial pollution in the O-ring adjacent to the shower head was observed. A brownish deposit was observed on the connection spot (the surface of the O-ring) after the removal of the shower head. The spot represented the path of tap water passed to the consumers. The highest numbers of microbes from those surfaces were 422 and 455 cfu/cm^2^, at a home and school, respectively, indicating a possible pollution source that deteriorated tap water quality (Table 2). The HPCs from the surface were 224 and 301 CFU/cm^2^ for homes and schools, respectively, from each of 10 units. Four home and five school units had HPCs over 300 CFU/cm^2^. The HPCs from the shower heads in the schools were higher than the HPCs from homes. The household water plumbing line is close to the tap the consumer uses for drinking water. However, the regular management of the facilities is not positively performed because most building management depends on consumers to replace and repair facility components. Although shower heads from residential areas were initially examined to detect the existence of *Legionella*, it was not detected in the shower heads. However, Lee detected *Legionella* in tap water in several samples taken from Korean pubic spas [24]. Hsu *et al.* reported that hot tubs studied were generally set up in the closed and semi-closed spaces. These provide ideal conditions for *Legionella* infections because human infection occurs through the inhalation of aerosols generated by the hot water contaminated by *Legionella* [26]. Kim *et al.* concluded that *Legionella* was present in samples of hot water, taps, and shower heads in Gwanju city, Korea, and identified *L. pneumophila* serogoroup 1 as a predominant species. In particular, six of 62 samples taken from hot shower water in hospitals contained *Legionella* [27].

**Table 2:** HPCs per unit surface area in deposits under the shower head.

Home	CFU/cm^2^	School	CFU/cm^2^
H1	48	S1	88
H2	120	S2	185
H3	389	S3	322
H4	422	S4	281
H5	145	S5	455
H6	100	S6	392
H7	144	S7	430
H8	101	S8	395
H9	398	S9	196
H10	370	S10	267

Samples H1, H2, S1, and S2 were taken from cold-water showers only. However, the other samples were taken from shower heads that supply both hot and cold water together.

In Korea, shower heads are more often used in homes than the divided type of hot and cold water taps. Yoon and Lee reported that residual chlorine was depleted at the end of the distribution system, and they formulated a relationship between bacterial regrowth and the residual chlorine in the water distribution system [1]. This result indicated that various circumstantial variations in tap water, such as residual chorine depletion and nutrition intrusion over a long residual time in pipes and water facilities, such as a reservoir tank, when water flows through a supply system. Hence, it has been shown that tap water quality can deteriorate before it reaches the tap, even though the water quality initially produced in the water plant meets the required standard.

Various bacteria were identified on shower heads from residential areas, as presented in Table 3. Three *Microbacterium* species, *Microbacterium lacticum*, *M. lacus*, and *M. oxydans*, were identified. Funke *et al.* reported that *Microbacterium* infections are rare in humans. However, infections with these species have more frequently been observed in immunocompromised patients [28]. It is thought that shower heads are a possible pathogen habitat, as they accumulate deposits. *Rhodococcus corynebacterioides* was identified in this research. The first report of sepsis by *R. corynebacterioides* infection in a human was made in a 64-year-old high fever patient with myelodysplastic syndrome [29]. *Staphylococcus* *warneri* is rarely known to cause diseases such as bacteremia, but it can cause infections in immunocompromised patients [30].

**Table 3:** Identification of bacteria on shower heads.

Sample	Microorganisms	Sample	Microorganisms
B1	*Acinetobacter* *guillouiae*	B6	*Acidovorax* *temperans*
			*Sphingobium* *lactosutens*
	*Acidovorax* *temperans*		
	*Sphingomonas* *paucimobilis*		
B2	*Stenotrophomonas* *maltophilia*	B7	*Chryseobacterium* *hominis*
			*Sphingobium* *lactosutens*
	*Acinetobacter* *guillouiae*		*Methylobacterium* *aminovorans*
			*Stenotrophomonas* *maltophilia*
	*Acidovorax* *temperans*		
B3	*Stenotrophomonas* *maltophilia*	B8	*Stenotrophomonas* *maltophilia*
	*Gordonia* *sputi*		*Sphingomonas* *paucimobilis*
	*Microbacterium* *lacticum*		
	*Microbacterium* *lacus*		*Acinetobacter* *guillouiae*
B4	*Acidovorax* *delafieldii*	B9	*Staphylococcus* *warneri*
			*Pseudoxanthomonas* *mexicana*
	*Sphingomonas* *koreensis*		*Agromyces* *soli*
			*Rhodococcus* *corynebacterioides*
	*Chryseobacterium* *defluvii*		*Sphingopyxis* *chilensis*
	*Stenotrophomonas* *maltophilia*		
B5	*Sphingobacterium* *siyangense*	B10	*Acidovorax* *temperans*
			*Sphingomonas* *paucimobilis*
	*Sphingomonas* *koreensis*		
	*Sphingomonas* *ginsenosidimutansMicrobacteriumoxydans*		

*Legionella* was not detected on any of the 10 shower heads tested.

In this study, the predominant bacteria found in home shower heads were *Stenotrophomonas maltophilia*, *Sphingomonas paucimobilis*, and *Acidovorax temperans*. *S. maltophilia* is not known to be highly contagious, but this bacterium is capable of opportunistic infection, and it is also an important nosocomial pathogen in immunocompromised patients and causes significant patient mortality [31]. Furthermore, entire hospital populations could be affected by water polluted with the opportunistic pathogen in the hospital’s drinking water system, with catastrophic results. *S.* *maltophilia* is well known to form biofilm on surfaces, such as plastics. Bacteria grown in biofilms on water systems were observed to be resistant to chlorine [32]. *S. maltophilia* is an obligate aerobic bacterium and shows multi-drug resistance [31,33]. This bacterium was identified on shower heads in five places.

*Sphingomonas* *paucimobilis* is also an obligate aerobic bacterium associated with nosocomial infections. *S. paucimobilis* is known to be resistant to specific antibiotics [34]. Lin *et al.* concluded that *S. paucimobilis* might cause various infections in healthy as well as immunocompromised patients [35]. Its capacity to degrade hydrocarbons and plasticizers has also been reported [36]. This finding indicated that bacterial multiplication is related to the decomposition of the plastic material used in water systems and the problem of waterborne diseases caused by this bacterium. Additionally, the existence of both *Stenotrophomonas maltophilia* and *Sphingomonas paucimobilis* in biofilms should be evaluated in relation to the decrease in the chlorine susceptibility in the Korean water treatment system. *Acidovorax temperans* is an abundant member of activated sludge. *A. temperans* strains CB2 is known to adhere readily to surfaces, where it forms biofilms [37].

*Microbacterium* *lacticum* is a thermoduric, opportunistic microorganism that is heat-resistant, so it has a high probability of growing in a hot water supply. The temperature at which *M. lacticum* dies was measured at 73 °C [38]. Selecting water treatments and processes, such as food manufacturing, should be carefully considered. The multiplication of *M. lacticum* in water supply systems can inhibit the management of drinking water because the prevention of bacterial counts cannot be guaranteed after treatment by boiling. These predominant bacteria have strong resistance levels to heat or antibiotic agents and are opportunistic microorganisms. Hot tap water contaminated by these microorganisms can also be transmitted in aerosols generated by shower heads. Various routes of infection other than tap water should be considered, including aerosol generated through microbially contaminated shower heads.

### 3.4. Migration of VOC from Plastic Pipes

The experiment measured the VOC levels that migrated from five types of plastic pipes, PB, PVC, PP, PE, and cPVC, as presented in Table 4. Skjevrak *et al.* reported that VOCs in biofilms inside high-density polyethylene (HDPE) pipes contributed to an off-flavor in drinking water [19]. Styrene, which may be toxic to the gastrointestinal tract, kidneys, and respiratory system, was detected in all pipes tested. It has been previously reported that styrene was not observed in seven water treatment plants in Korea [39]. In this study, the styrene level detected was not high enough to constitute a health hazard.

**Table 4:** VOC substances in plastic pipes (unit: μg/L).

Items	PB	PVC	PP	PE	cPVC
Vinylacetate	1.2	0.0	0.7	0.8	0.9
*N,N*-Dimethylaniline	0.4	0.5	0.5	0.3	0.3
*cis*-1,2-Dichloroethylene	0.4	0.0	0.1	0.4	0.3
Epichlorohydrin	0.4	0.3	0.7	0.3	0.4
Styrene	0.3	0.3	0.4	0.4	0.2

Carbon tetrachloride, vinylchloride, acrylonitrile, 1,1,1-trichloroethane, 1,3-butadiene, 1,2-butadiene, 1,1-dichloroethylene, dichloromethane, 1,1,2-trichloroethane, and tetrachloroethylene were not detected.

In this study, *cis*-1,2-dichloroethylene was detected in PB, PP, PE, and cPVC pipes. It was thought that in the worst scenario, consumers could be affected by breathing the vapors of *cis*-1,2-dichloroethylene while bathing, dishwashing, and cooking with water from a polluted water system. Levels of epichlorohydrin, a known carcinogen, from PP pipes were relatively high compared to other plastic pipes [40]. The styrene level was 0.3 μg/L in PB and PVC pipes. Problems with taste and odor caused by PVC hose have been reported for tap water [41]. Styrene and vinyl acetate have been known to have specific tastes. Heim reported that polymer-based pipe material played an important role in the occurrence of odor and TOC levels in water. PEX pipe showed a higher growth of coliform bacteria than copper pipe [42].

The levels of released metal from plastic pipes were monitored in five pipe systems, PB, PVC, PP, PE, and cPVC. The metal levels in new plastic facilities were not serious except for PVC and cPVC, respectively. 6 μg/L of tin and 2.7 μg/L of lead were detected in new cPVC and PVC pipe in this experiment (data are not shown). Sadiki and William concluded that organotin compounds were found in 29% and 40% of distribution water in 28 and 21 sites, respectively, in new PVC pipes typically less than 6 months old during winter-spring and autumn [14]. However, the compounds were not detected after 15 days of operation. Al-Malack reported that UV radiation expedited the migration of lead from PVC pipe [43]. When water facilities with PVC pipes on the ground and PVC water reservoirs situated on rooftops are exposed to UV irradiation, the leaching concentration could be affected.

**Figure 5:**
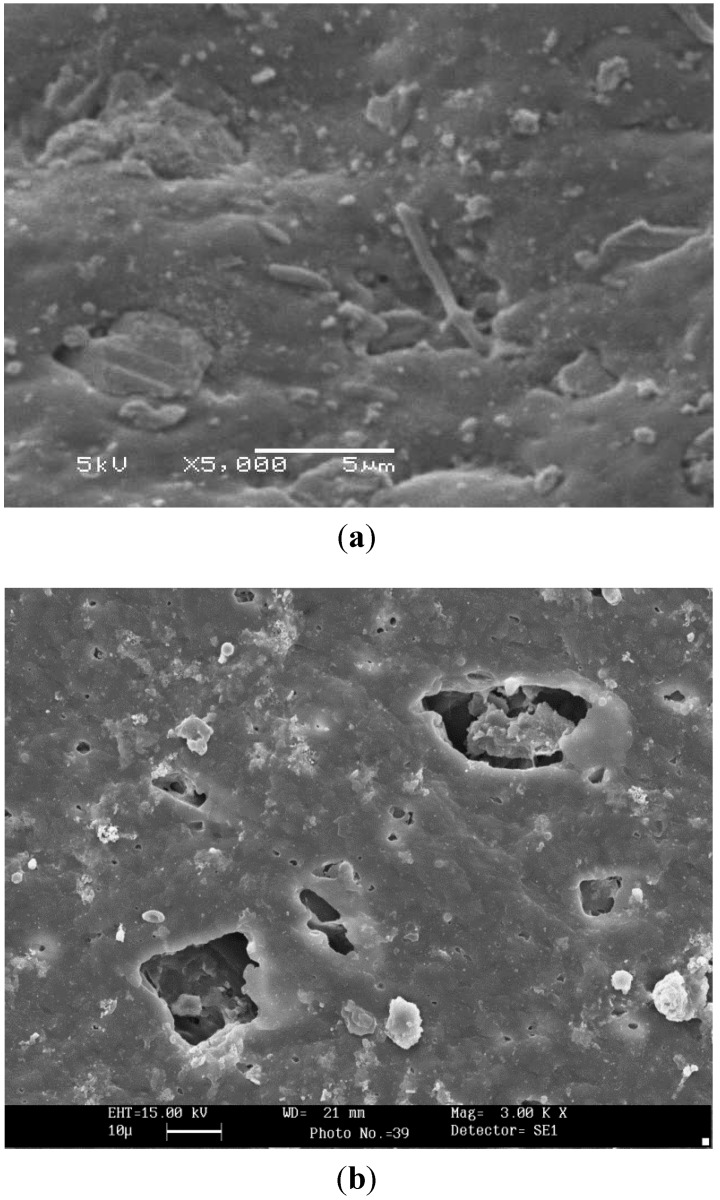
SEM photo of PVC pipes. (**a**) PVC pipe surface with tap water after one year of operation; (**b**) PVC pipe surface with autoclaved tap water after one year of operation.

As shown in Figure 5, the surface of the PVC pipe was not smooth after 360 days of operation with tap water. In addition, deposits on the surface of the pipe were observed, in addition to damage and pipe material loss. These effects might be occurred as a result of the leaching of the pipe material in PVC pipes and the subsequent weakening in pipe strength from pipe material loss.

### 3.5. Effects of Chlorine and Membrane Filtration on Biofilms

**Figure 6:**
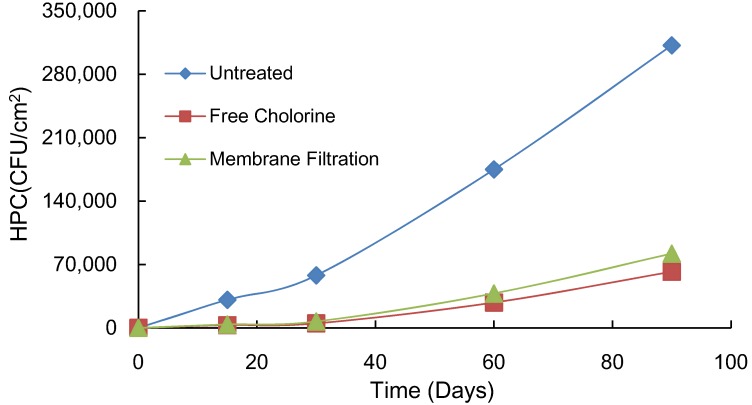
Comparison of biofilm control methods in stainless steel pipe.

The control methods, chlorine disinfection and membrane filtration, of biofilm accumulation on stainless steel pipe as assessed by HPC are compared in Figure 6. Biofilm levels in untreated conditions increased linearly in terms of HPC with time. The use of stainless steel pipe in the water supply system has increased in Korea because of its low corrosion rate and capacity to provide a hygienic and aesthetically stable supply of tap water. However, in experiments using a glucose solution, Lee reported that the rate of biofilm formation was the highest on stainless steel when compared to galvanized and copper pipes [44]. In this experiment, attached HPCs on stainless steel pipes were 20% of the level in the control with a chlorine dose of 4 mg/L. Kim *et al.* reported that half of the chlorine dose was removed in 25 h at 25 °C in tap water in a bottle test. In addition, they suggest that residual chlorine could distribute evenly from water plant to water reservoir at the end-point of the distribution system by rechlorination, and the highest dose of chlorine for rechlorination was 6 mg/L, considering the formation of trihalomethanes (THMs) in a water plant and a water distribution system located in “*D*” city, Korea [45].

This study showed that the microfiltration method could control attached HPCs as effectively as chlorine. Chlorine in tap water can increase AOC levels [46]. In particular, the reduction of chlorine susceptibility may contribute to the germicidal effect, and its constant application increases resistance to chemicals. The availability of nutrients could change with variations in the physical status, such as treatment methods, applied to the tap water supply. The increase in nutrient levels could affect the multiplication of microorganisms in the water supply system. After treatment by microfiltration, HPCs attached to the pipe were 26% of the control at 90 days. Lee reported that physical operations, such as pressurized water flushing and chlorine addition, would be useful in controlling biofilm growth in the water supply system [20].

## 4. Conclusions

The following conclusions can be drawn from this study:

(1) The results of the pipe coupon test indicated that biofilm accumulation in pipes is stimulated by adding AOC and inorganics supplement. However, biofilm formation was significantly greater for AOC than for inorganics supplement in stainless steel pipe. Bacterial growth in biofilms was stimulated by the heating of the tap water. Based on these results, the removal efficiency of nutrient residuals through the treatment processes in water treatment plants may affect biofilm formation in the distribution system. Both chlorination and microfiltration reduced biofilm formation, as measured by HPCs on pipe walls.
(2) After inspection of the internal surfaces in PVC systems, significant pipe loss was observed on pipe surfaces after 1 years of operation, and VOC substances, such as vinylacetate, NN-DMA, *cis*-1,2-dichloroethylene, epichlorohydrin, and styrene were identified in five kinds of plastic pipes (PVC, PB, PP, PE, and cPVC).
(3) The predominant microorganisms on household shower heads taken from “*N*” city included *Stenotrophomonas maltophilia*, *Sphingomonas paucimobilis*, *Acidovorax temperans*, and *Microbacterium lacticum*. However, *Legionella* was not found. Some species detected are recognized as opportunistic and potential human pathogens in causing nosocomial infection, such as *Stenotrophomonas maltophilia* and *Sphingomonas paucimobilis.* The existence of these microorganisms in the water supply system could cause considerable risks to immnocompromised people. In these cases, shower heads may act as possible pollutant sources for the microbial contamination of water. Further research is required to characterize the persistence of *Stenotrophomonas maltophilia* and *Sphingomonas paucimobilis* in the presence of water treatment, including chlorine disinfection, of the water distribution system.
